# Smart Fuzzy Petri Net-Based Temperature Control Framework for Reducing Building Energy Consumption

**DOI:** 10.3390/s23135985

**Published:** 2023-06-28

**Authors:** Wael Deabes, Kheir Eddine Bouazza, Wasl Algthami

**Affiliations:** 1 Department of Computer Science in Jamoum, Umm Al-Qura University, Makkah 25371, Saudi Arabia; wadeabes@uqu.edu.sa; 2 Computer Information Science Division, Higher Colleges of Technology, Abu Dhabi P.O. Box 25026, United Arab Emirates; kbouazza@hct.ac.ae

**Keywords:** petri net, fuzzy logic, fuzzy petri net, smart building, temperature control, energy consumption

## Abstract

This study addresses the pressing issue of energy consumption and efficiency in the Kingdom of Saudi Arabia (KSA), a region experiencing growing demand for energy resources. Temperature control plays a vital role in achieving energy efficiency; however, traditional control systems may struggle to adapt to the non-linear and time-varying characteristics of the problem. To tackle this challenge, a fuzzy petri net (FPN) controller is proposed as a more suitable solution that combines fuzzy logic (FL) and petri nets (PN) to model and simulate complex systems. The main objective of this research is to develop an intelligent energy-saving framework that integrates advanced methodologies and air conditioning (AC) systems to optimize energy utilization and create a comfortable indoor environment. The proposed system incorporates user identification to authorize individuals who can set the temperature, and FL combined with PN is utilized to monitor and transmit their preferred temperature settings to a PID controller for adjustment. The experimental findings demonstrate the effectiveness of integrating the FPN controller with a convertible frequency AC compressor in significantly reducing energy consumption by 94% compared to using the PN controller alone. The utilization of the PN controller alone resulted in a 25% reduction in energy consumption. Conversely, employing a fixed-frequency compressor led to a 40% increase in energy consumption. These results emphasize the advantages of integrating FL into the PN model, as it effectively reduces energy consumption by half, highlighting the potential of the proposed approach for enhancing energy efficiency in AC systems.

## 1. Introduction

Energy consumption is a crucial aspect of economic development and sustainability worldwide. The KSA has one of the highest energy-consumption rates in the world due to rapid economic and population growth. Air conditioning use is a major contributor to this trend, especially in the western region of the country, where temperatures can exceed 50 °C during the summer months [1]. In this western region, which has the highest level of electricity consumption in the country, electricity is used by 99% of energy consumers, with the majority (80%) consuming less than 4000 kWh per month and a small percentage (1.4%) consuming more than 10,000 kWh per month [1,2].

**Figure 1:**
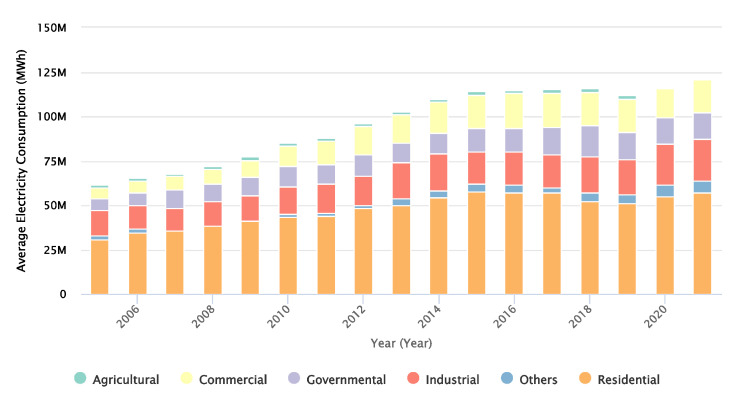
Electricity consumption in the KSA from 2005 to 2020 [3].

According to the International Energy Agency (see Figure 1), electricity consumption in Saudi Arabia has increased by more than 50% between 2007 and 2017 [3]. Indeed, the use of air conditioners is responsible for more than 70% of peak electricity demand during the summer months in Saudi Arabia [3]. This high level of energy consumption has created several challenges for the country.

The way energy is currently consumed in buildings is not suitable for the future. As a result, researchers and engineers are exploring different technologies and strategies to decrease energy usage while still providing comfort and well-being to occupants. In order to improve energy efficiency in the KSA and optimize energy consumption, recent studies have explored the use of intelligent building control systems using PNs.

Petri described PN theory as a network-like model used in research on automated communication that is relevant to the field [4]. PNs have emerged as a powerful tool for modelling and analysing systems in various fields such as computer science, engineering and biology. They are characterized by their simplicity, flexibility, and ability to handle concurrency, making them suitable for a wide range of applications [5].

In recent years, researchers have explored new directions for PNs, such as their use in modelling artificial intelligence systems and Big Data analysis. Researchers have developed several extensions and tools from PNs to model complex systems and analyse large-scale models, such as hierarchical and coloured PNs, as well as model checking techniques.

The basic theory of PNs and their formal properties, including the concepts of bounding and integrity are presented in [6]. The authors in [7] presented the extension of coloured PNs, allowing to model and control more complex data structures and systems with multiple interacting objects. The authors in [8] introduced hierarchical coloured PNs, allowing modular and hierarchical modelling of complex systems. These papers have contributed significantly to the development and use of PNs in computer science and related fields, advancing our understanding of complex systems and their analysis techniques.

In the last two decades, there has been increasing interest in the use of PNs in building control applications. The authors in [9] developed a hybrid PN model for building heating, ventilation, and air conditioning (HVAC) systems and proposed a control strategy based on the model. They demonstrated the effectiveness of their approach using a case study. A PN-based modelling approach for smart home appliances enabling a better understanding of their behaviour and interactions was proposed in [10]. The proposed approach was applied to a smart washing machine and a smart dryer in a smart home environment. The PN models were able to capture the behaviour of the appliances and their interactions with the smart home environment.

In [11], a PN-based framework for reducing building energy consumption by optimizing the temperature control was developed. The PN model incorporated real-time weather conditions and occupancy patterns to adaptively adjust the temperature set-points of HVAC systems. The results showed significant energy savings and improved thermal comfort for occupants. Authors in [12] proposed an intelligent PN-based system to control and monitor the greenhouse temperature. The system utilizes wireless sensors to collect temperature and humidity data and control actuators to adjust the temperature in real-time. The results showed improved temperature control and energy efficiency, leading to higher crop yields. In [13], the authors presented a design and implementation of a smart home system and a self-control window using a field programmable gate array (FPGA) and PN modelling techniques. The self-control window uses a PN model to detect changes in the environment and adjust its opening and closing accordingly.

In [14], a PN was used to verify the feasibility and soundness of the system model and optimize its performance by eliminating improper states. The system aims to enhance safety and prevent property damage by enabling real-time monitoring and control of home appliances using IoT and machine learning technologies.

Fuzzy logic (FL) was first introduced by Lotfi Zadeh in 1965 [15] as a mathematical framework for dealing with uncertainty and imprecision. FL allows the representation of a partial truth, useful in situations where information is incomplete or uncertain [16]. In the last two decades, FL has gained significant popularity and has been widely utilized in numerous studies. For instance, the authors in [17] employed FL in the development of an optimal energy management strategy for a hybrid power unmanned aerial vehicle (UAV) that integrates fuel cells and battery systems. A FL-based emulated inertia control was implemented in [18] in a supercapacitor system to enhance inertia in a low-inertia grid with renewable energy sources.

FPN is a combination of FL and PN. Indeed, FPN is a formalism that inherits the graphical and mathematical foundations of the PN model, and it is used to construct, calculate, and make inferences in expert systems containing fuzzy data. FPN was originally proposed by Zimmermann in [19]. FPN enables the modelling of complex systems with imprecise and uncertain information [20]. Since their introduction, FPNs have been used in a variety of applications, including control systems, manufacturing systems, and decision support systems. They have also been extended in various ways, such as the use of interval-valued fuzzy sets or the incorporation of timed data.

In [21], the author applied FPNs to the control of complex industrial processes. They demonstrated how FPNs handle imprecise data and provide more accurate results. The paper highlighted the potential of FPNs as a tool for modelling and controlling complex systems. The authors in [22] discussed various aspects of FPNs, such as their syntax and semantics, graphical representation, analysis and verification techniques, and applications in areas such as control, diagnosis, and scheduling. They also highlighted some of the advantages and challenges of using FPNs, such as their ability to capture both qualitative and quantitative aspects of a system, but also their potential complexity and computational cost. In [23], a comprehensive review of the application of FPNs in knowledge representation and reasoning is described. The authors presented various examples of FPNs in action, including their use in medical diagnosis, fault diagnosis, and process control. In [24], a robust and efficient system for decision making and control, in the GEMMA guide paradigm, was developed, utilizing FPNs as a modelling tool. The applications of FPNs in various domains such as control, optimization, and fault diagnosis and their recent advances and extensions, such as hybrid FPNs that combine fuzzy and crisp information, and dynamic FPNs that can model systems with changing structures or behaviour are discussed in [25].

The motivation of this work is driven by the increasing demand for energy resources and the need for energy efficiency, particularly in countries such as the KSA. Energy consumption in buildings, particularly for air conditioning (AC) systems, represents a significant portion of the overall energy usage. Therefore, there is a pressing need to develop intelligent and energy-saving frameworks that can optimize energy use while ensuring a comfortable environment for occupants.

In [11], a regular PN was utilized for temperature control based on a user-defined temperature. However, recognizing the subjectivity and variability of individual preferences for temperature, we have enhanced the framework by integrating FPNs. By considering fuzzy concepts such as “good temperature”, “warm temperature”, or “low temperature”, the system can accommodate the subjective and varying preferences of different users, providing a more personalized and adaptable solution. This integration allows for the modelling of uncertainty and imprecision, enabling a more effective approach to address diverse user preferences, ultimately improving user comfort and satisfaction. Furthermore, by optimizing energy consumption based on firing degrees within the FPN framework, the proposed approach offers significant improvements over the previous model. Through this advancement, we aim to provide a more effective and user-centric temperature control system that achieves both energy efficiency and occupant comfort.

The main goal of this paper is to explore the potential of FPNs in reducing energy consumption and promoting sustainable energy practices in smart buildings in the KSA. We propose a framework based on three different stages. The initial phase involves identifying the user through a user identification sub-system, and obtaining their preferred temperature. In the subsequent phase, an FPN utilizes the data gathered in the first stage to create the desired temperature patterns for the users and communicates it as a reference signal to the following stage. The third stage involves a regulatory process utilizing a PID controller, which synchronizes the actual room temperature with the preferred temperature generated by the user.

The paper is structured in the following manner: Section 2 introduces FPNs, while Section 3 discusses FL and its application in this paper. The PID controller and user identification processes are explained in Section 4 and Section 5, respectively. In Section 6, the FPN-based smart temperature control framework developed in this paper is described. The experimental work conducted to test the framework is covered in Section 7. The advantages and effectiveness of the developed framework are discussed in the subsequent two sections. Finally, the conclusion summarizes the accomplishments of the proposed approach.

## 2. Fuzzy Petri Nets (FPNs)

### 2.1. Petri Nets (PNs)

**Figure 2:**
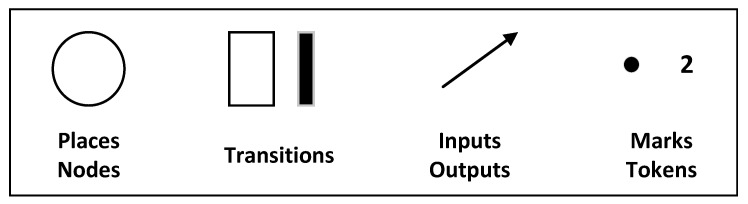
PN elements with their representation [26].

A PN or a place/transition net is a group of directed arcs connecting places and transitions [26]. It was first introduced by Dr. Carl Adam Petri in 1962 [4]. It is a formal specification method for multitasking and synchronization. It consists of places, arcs and transitions as shown in Figure 2 with their different representation.

One place may have a token or not. These types of tokens can be used as a demonstration of the current system status [23]. The default capacity of an arc is one, but if the capacity is more than one, it is explicitly mentioned. The arcs have conditions that describe the action or trigger that causes the transition. Places have infinite capacity by default, while transitions do not have any capacity and cannot store tokens. Therefore, arcs can only connect places to transitions and vice versa. A PN can be characterized by five values, including a numerical representation of the places, a numerical representation of the transitions, an explanation of the relationship between the first two values, a weight function, and the initial sign of the PN.

**Figure 3:**
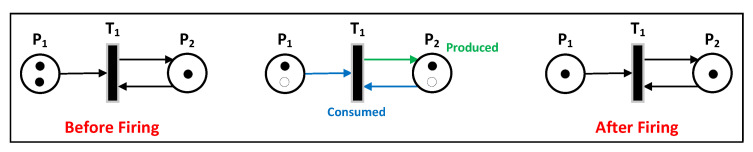
PN tokens produced and consumed [26].

PNs are a type of diagram used to depict data flow, but with a unique syntax that is more closely related to multitasking. Transitions are fired when all their inputs have a token, and they are triggered by events such as clocks or synchronization functions. Tokens are consumed from one place and moved through transitions before being produced in another place. When the sum of tokens from all inputs is at least equal to the weight of the arc from that input to the transition, the transition is activated. If the weight of the arcs and the capacity of the output places allow this, the tokens in the input are transferred to the output when the transition is successful. Input places consume tokens, while output places produce tokens through the firing of transitions as shown in the Figure 3.

**Figure 4:**
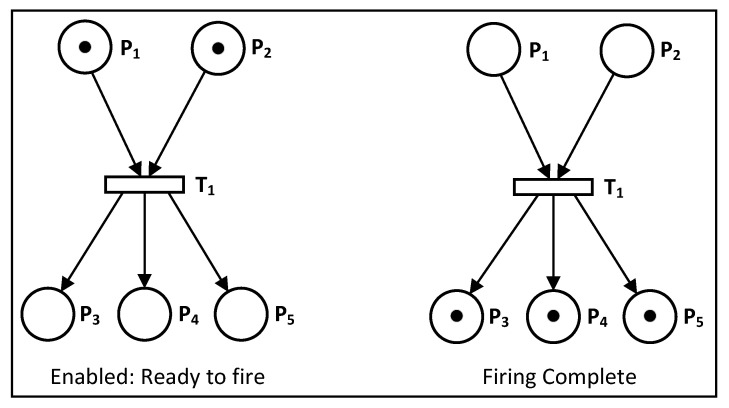
PN ready to fire and fire complete with the arcs’ weight as one [26].

The connection between the input and output places in PNs is influenced by the weight of the connecting arc. This weight determines how tokens are distributed in the output. Typically, the arcs have the same weight, which is set to one by default, as mentioned earlier. Figure 4 provides another example of a transition being fired with arcs of equal weight, each set to one.

### 2.2. Fuzzy Logic (FL)

FL refers to a mathematical framework that provides a way to reason with uncertain or imprecise information [15]. It is a powerful tool that has been used to model complex systems where precise rules are difficult to define. FL has found a wide range of applications in various fields, such as engineering, computer science, finance, and medicine, due to its ability to handle complex systems with incomplete or uncertain information [27]. In recent years, FL has gained significant interest as a method for solving various complex problems, such as temperature control [28].

FL has been particularly useful in the field of temperature control due to the complexity and non-linearity of the control systems [29]. Traditional temperature control systems rely on mathematical models and rule-based algorithms, which are often imprecise and cannot capture the full complexity of real-world systems. However, FL-based temperature control systems use linguistic variables to create a more intuitive representation of the control rules. These systems create a set of fuzzy rules to describe the system’s behaviour by fuzzifying the input variables and defuzzifying the output variables to obtain a crisp control signal. Recent studies have shown that FL controllers have several advantages over traditional control systems in temperature control applications [29]. FL-based controllers can handle complex systems with non-linear behaviour and can be easily tuned to achieve the desired performance metrics. Additionally, they are robust to disturbances and can maintain stable control even in the presence of uncertainties and noise.

Numerous applications of FL controllers in temperature control have been reported in the literature. For instance, FL controllers have been used in the temperature control of refrigeration, HVAC, and the thermal management of electronic systems [30]. FL controllers have also been employed to control the temperature of buildings by integrating data from multiple sensors and actuators. Overall, the use of FL in temperature control systems provides a promising approach to addressing complex and non-linear control problems.

FL involves the concept of precisiation, which is distinct from representation and operates to a higher degree. When working with linguistic variables, they can be treated as fuzzy sets with a certain number of membership functions, and FL can be used to calculate these values. The term precisiation refers to the replacement of numbers with words, which can be justified in various ways. Firstly, words can be used when numerical values are unavailable to describe a variable. Secondly, words are preferable in dealing with imprecise matters where cost-cutting or a decision needs to be made. Finally, using words in calculations can aid in explaining complex fuzzy concepts that have been precisiated [27]. The use of FL is essential in replacing numbers with words and making the precisiation of these concepts possible.

### 2.3. Fuzzy Petri Nets (FPNs)

**Figure 5:**
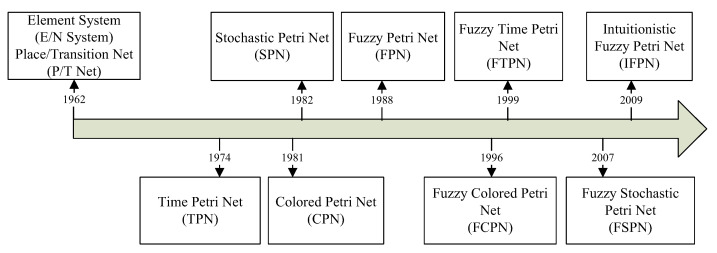
Development of the PN model types [21].

FPNs are an extension of PNs that use FL to represent and handle uncertainty and imprecision in the modelling of systems. In [21], the author utilized FPNs to overcome the shortcomings of conventional PNs in managing vague information and coping with uncertainty in knowledge-based systems (KBS) or systems with uncertainty. Figure 5 illustrates the evolutionary process of PNs leading to FPNs. FPNs combine the graphical representation capability of PNs with the uncertainty handling capability of fuzzy set theory. FPNs use FL to represent the firing rule of a transition in a PN, where the transition firing is a fuzzy event. The fuzzy rule is represented by a membership function that maps the degree of membership of a fuzzy set to the degree of transition firing [31].

FPNs have been successfully applied in various fields, including control systems, manufacturing systems, transportation systems, and communication networks. They have been used to model complex systems with uncertainty and imprecision, such as fuzzy control systems, fault diagnosis systems, and decision-making systems [25]. FPNs have also been used in the design of intelligent systems, such as intelligent transportation systems and intelligent manufacturing systems. FPNs also have the ability to model complex interactions between multiple subsystems, making them useful for the design of large-scale systems. In addition, FPNs can be easily integrated with other techniques, such as artificial neural networks and genetic algorithms, to enhance their capabilities [32].

To apply FPNs, there are three stages needed, as outlined in [20]. The first stage involves creating FPN models for any system that has a level of uncertainty associated with it. The second stage is to understand the different aspects related to the application that FPN is used with to reach a point where an algorithm can be established. The last stage focuses on the implementation of the designed algorithm and dealing with its appropriate parameters.

To construct an FPN, a number of actions need to be taken. Initially, data about the system must be gathered from either an expert user or a database. Once this data has been acquired, it must be fuzzified into a fuzzy set. Following this, the PN must be established, which entails developing the rule base, inference base, and outcome defuzzification. There are various methods to create the rule inference, such as norms. The selection of a method depends on the specific needs of the FPN-based system being created [33,34].

The FPN methodology was also applied in the electricity sector to achieve power supply stability in case of abnormalities such as short circuits, as demonstrated in a study conducted in Taiwan that utilized IF-THEN rules along with AND and XOR [35]. Another study analysed a paper mill using an FPN and found its outcomes to be encouraging when compared to conventional techniques [36]. An FPN was also used to detect errors and analyse complex computer-controlled systems in a study conducted by [37]. In all of these studies, FPN models were utilized and their outcomes were compared to other techniques to validate their effectiveness. Section 6 will provide a more detailed explanation of how FPNs are implemented.

## 3. PID Controller

PID controllers are widely used in the field of process control to maintain desired levels of process variables such as temperature, flow rate, pressure, and speed. PID controllers are considered effective and reliable because they are easy to implement and tune. The acronym PID stands for proportional, integral, and derivative, which are the three control modes employed by these controllers. Proportional control adjusts the output in proportion to the error, while integral control accounts for accumulated errors over time, and derivative control adjusts the output based on the rate of change of the error [38]. They work by continuously monitoring the process variable, comparing it to a set-point, and adjusting the output signal to bring the process variable back to the set-point. The controller calculates the error between the set-point and the actual process variable and then applies proportional, integral, and derivative terms to the output signal to correct for the error and stabilize the process. Due to their high accuracy and stability, PID controllers are commonly used in various industries such as manufacturing, chemical processing, and energy production. In fact, more than 90% of all controllers used in industry are PID controllers [39].

## 4. User Identification

To apply energy-consumption-reduction techniques to a building’s rooms, the system needs to identify and authenticate the users who have access to the room and are able to set their preferences. There are three main methods that can be used for user identification and authentication: knowledge-, token-, and biometric-based methods. The first method, knowledge-based, requires the user to provide a piece of information that only they would know, such as a password or PIN. The second method, token-based, involves the use of a physical object, such as an ID card or a smart card, that the user must possess in order to gain access to the room. The third method, biometric-based, uses the unique physiological or behavioural characteristics of the user, such as fingerprints or facial recognition, to authenticate them [40].

Facial recognition is a biometric-based method of user identification and authentication that has gained a lot of attention in recent years. It involves using computer algorithms to analyse and compare patterns of facial features in an image or video to identify or verify a person’s identity. Facial recognition technology has numerous applications, including security systems, mobile devices, payment systems, and access control systems. For example, airports may use facial recognition technology to identify passengers as they go through security, or companies may use facial recognition to verify employee identities for access to secure areas or sensitive information. Facial recognition technology continues to evolve and improve, with developers working to address the technical and ethical challenges associated with its use [41].

## 5. Smart FPN-Based Temperature Control Framework

In this work, we developed a system that uses FPN to regulate the temperature of AC systems based on user preferences. The FPN used in the system enables modelling of complex systems that involve uncertain and ambiguous data. The decision-making mechanism of the FPN system receives data from different sources, such as the user preference model, the temperature prediction system, and the energy reduction system, to determine the ideal temperature set-point for the AC unit. This method reduces energy consumption and improves user satisfaction by considering an individual’s preferences. In addition, the integration of FL into different aspects of the system allows the system to deal with ambiguous and uncertain data, improving its versatility and adaptability to different scenarios.

**Figure 6:**
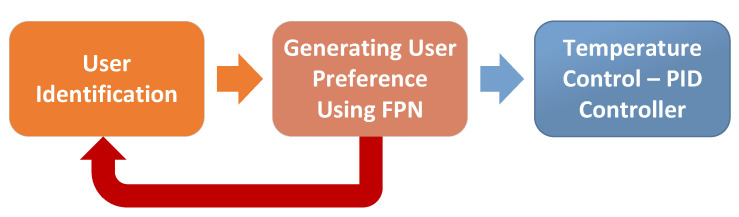
Temperature control framework.

The framework consists of three stages, as shown in Figure 6, each dedicated to a specific task.

1. User identification sub-system;
2. FPN;
3. PID controller.

The most significant aspect of this work is the integration of several stages, including person identification, FPN-based monitoring, and temperature control using a PID controller. The framework combines the advantages of each component, making it feasible, easy to implement, and very flexible. In addition, it can be easily modified and customized to meet different requirements, such as incorporating additional sensors or temperature control algorithms. The framework can also be integrated with other systems to provide advanced features such as remote monitoring and control.

The initial stage of the framework involves identification of the user, the results are then passed to the next stage. The second stage generates the user’s preferred temperature based on the identification results, using FPN for modelling, supervision, and real-time implementation. The third stage uses a traditional PID controller to regulate the room temperature based on the user’s preferred temperature. The framework operates autonomously, monitoring the room and adjusting the temperature when users are present, and switching to sleep mode when no one is present to save energy. The most significant aspect of this work is the integration of several stages, including person identification, FPN-based monitoring and supervision and temperature control using a PID controller.

These three stages cooperate for the best experience for users and most importantly to control temperature and reduce energy consumption. In FPN, the whole set of places indicates the previous or next state of the system and may have a token bound to a degree of truth between 0 and 1 that represents the confidence in these values. FL can be used to define the transition firing conditions and the marking change in the FPN. In a traditional PN, the transition firing condition is based on a binary value (0 or 1) depending on the marking of the input and the firing rule. However, in an FPN the transition firing condition can be defined as a continuous value between 0 and 1, representing the degree of activation of the transition. This degree of activation is calculated based on fuzzy rules accounting for the marking of the input, the firing rule, and other relevant factors. Therefore, the integration of FL with PN is performed by setting the transition into a rule associated with a certainty factor value between 0 and 1. The certainty factor represents the strength of belief in the rule. In this research, we use FPN as a decision support system based on specific rules of the form: IF condition THEN action.

Arc weights in an FPN can also be fuzzy sets that represent the degree of membership between two nodes. The arc weight between a transition and a place can represent the degree to which firing that transition will fill or empty the place. The degree of membership is determined by FL, based on the firing condition and other relevant factors. For instance, a rule might specify that if the temperature is “very cold” and the user’s preference is “very warm”, then the transition to increase the temperature should fire with a degree of membership of 0.8. The rule’s fuzzy set defines the degree to which the condition is satisfied. Therefore, FL offers a strong method for dealing with uncertainty and imprecision in the PN. This allows for greater flexibility and reliability in controlling complex systems.

For example, consider an FPN that models a temperature control system. The inputs are the current temperature and the desired temperature, and the output is the control signal that regulates the temperature. The transition firing condition can be defined as a degree of activation that reflects the degree of match between the current temperature and the desired temperature. This degree of activation can be calculated using fuzzy rules that account for the difference between the current and desired temperatures, the rate of temperature change, and other relevant factors.

Once the degree of transition activation is calculated, it is used to determine the probability of firing the transition. This probability is then used to update the marking of the input and output. The marking of the inputs is decreased according to the firing rule, while marking the output is increased. The degree of transition activation is also used to calculate the degree of change in marking the output, reflecting the degree of control signal needed to regulate the temperature.

### 5.1. Room Model

The system response analysis for the AC would be performed in the Laplace domain (s-space), instead of the time domain. For an AC space of a fixed volume, the parameters could be illustrated as in Table 1.

**Figure 7:**
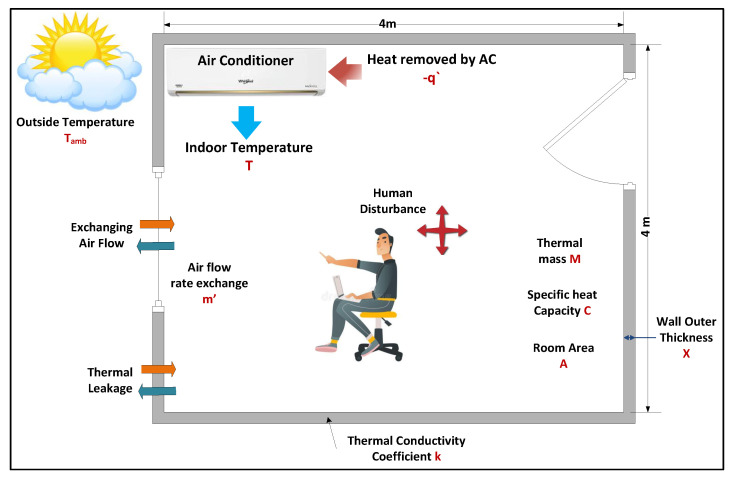
Temperature control mathematical model of the AC system [42].

The thermal mass includes the weights of the users and the thermal mass of the indoor air. Figure 7 presents the modelling of the temperature control in the AC space [42].

The equation representing the AC model is shown as follow:

(1) $$-\frac{dQ_{abs}}{dt}+\frac{kA(T_{amb}-T)}{X}=\frac{\partial }{\partial t}\oint_{cv}\;pud\forall -m^{\prime }C_{p}\left(T_{amb}-T\right)$$

where $m^{\prime }$ is the exchanging air flow, and $C_{p}$ is the heat capacity. Assuming $T_{amb}$ to be time-invariant, Equation (1) can be rewritten as:

(2) $$-\frac{dQ_{abs}}{dt}=M\cdot C\frac{d(T-T_{amb})}{X}+\left(m^{\prime }C_{p}+\;\frac{kA}{X}\right)\left(T-T_{amb}\right)$$

Taking the Laplace transform of Equation (2), the result is:

(3) $$-dQ_{abs}\left(s\right)=M\cdot C\cdot s\cdot T\left(s\right)+\left(m^{\prime }C_{p}+\frac{kA}{X}\right)T\left(s\right)$$

The transfer function model of the AC space is:

(4) $$-\frac{T(s)}{Q_{abs}\left(s\right)}=\frac{1}{M\cdot C\cdot s+m^{\prime }C_{p}+\frac{kA}{X}}$$

### 5.2. Sensor Model

**Figure 8:**
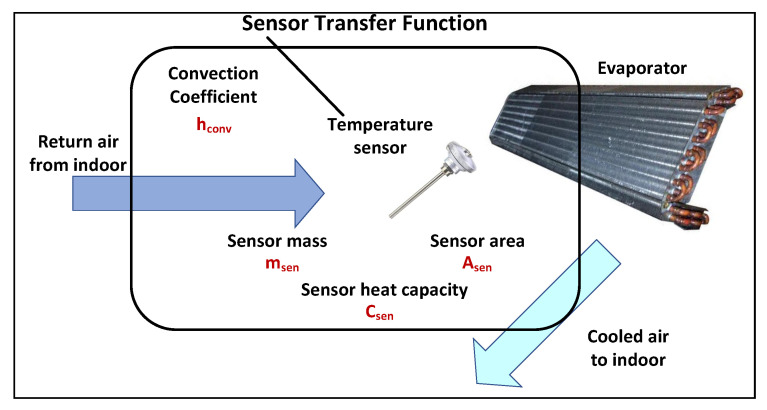
Temperature sensor installed on the evaporator of the AC.

To measure the room temperature and close the control loop, a high-quality sensor is used inside the indoor AC unit, placed in the centre of the evaporator as shown in Figure 8 [42].

The relationship between the return air temperature (*T*) and the measured temperature by the sensor ($T_{sen}$) can be written as:

(5) $$m_{sen}\;C_{sen}\;\frac{dT_{sen}}{dt}=h_{conv}\;A_{sen}\;\left(T-T_{sen}\right)$$

Taking the Laplace transform of Equation (5), the result is:

(6) $$\left(m_{sen}\;C_{sen}\cdot s+h_{conv}\;A_{sen}\right)\;T_{sen}\left(s\right)=h_{conv}\;A_{sen}\;T\left(s\right)$$

Therefore, the transfer function representing the model of the sensor is:

(7) $$\frac{T_{sen}\left(s\right)}{T(s)}=\frac{1}{\frac{m_{sen}C_{sen}}{h_{conv}A_{sen}}s+1}$$

### 5.3. Disturbance Model

The thermal leakage ($Q_{leakage}$) of the AC space can be approximated by the temperature difference of the indoor and outdoor space as:

(8) $$Q_{leakage}=\frac{kA(T_{amb}-T)}{X}$$

To maintain the air quality of the indoor space, according to the building technology rules, the minimum mechanical ventilation is 10 m^3^/h per unit surface area m^2^. The thermal leakage ($Q_{exchange}$) due to the exchanged air flow is:

(9) $$Q_{exchange}=m^{\prime }C_{p}\left(T_{amb}-T\right)$$

Regarding human disturbances inside the room, in order to maintain human functionality, body temperature has to be kept at a constant level. Therefore, thermal radiation from people (*n* person) affect the internal temperature of the room. Typically, a person releases about 70 W during sleeping and 100 W performing light effort. In this work, human disturbance is presented as Equation (10).

(10) $$Q_{human}=100*n$$

**Figure 9:**
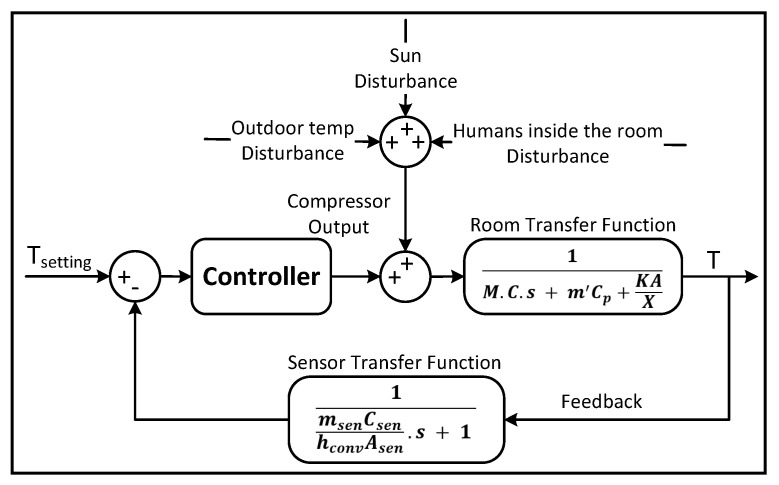
Air conditioner closed-loop system with disturbances.

By installing a temperature sensor in the evaporator of the indoor split-type AC unit to obtain a feedback signal, the open-loop AC space model becomes a closed-loop model, as shown in Figure 9.

The room transfer function of the AC space, sensor and controller are organized to simulate the responses of the indoor temperature and compressor output for the AC control design. The controller output relies on sensing differences between the setting point and real feedback signal to compute the power commands for either fixed- or convertible-frequency ACs. The compressor of the fixed-frequency AC is switched On or Off for a period of time, while the convertible-frequency AC changes the compressor output continuously to adjust the room temperature; both types are simulated later.

## 6. Experimental Work

### 6.1. AC Numerical Simulation

In order to simulate the AC model, we considered an office located on the third floor of the Al-Jamoum University College building, with an area of 16 m^2^. The used numerical data are presented in the Table 2.

Therefore, the room transfer function in Equation (4) is:

(11) $$\frac{T(s)}{-Q_{abs}\left(s\right)}=\frac{1}{2837s+12.82}$$

**Figure 10:**
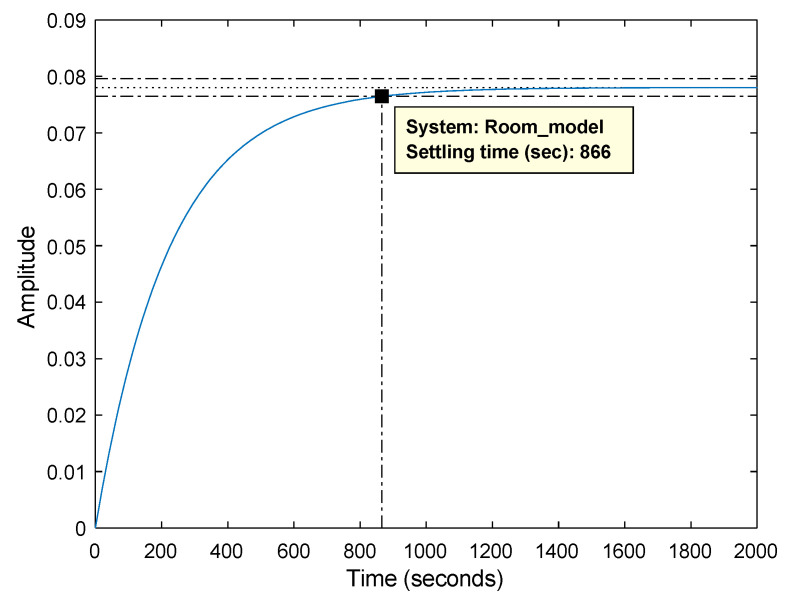
Step response of the room transfer function.

The step response of the room transfer function in Figure 10 shows that the room temperature is stabilized after 866 s (about 14 min), which is reasonable as thermal systems have slow dynamics. We can conclude from the steady-state gain that by adding 1 W to the room input, the temperature increases by 0.08 °C.

The sensor inside the AC is made from copper, aluminium or other metal composites, but they all have a response time of less than 30 s. For the purpose of this work, the chosen sensor possessed the characteristics listed in Table 3.

Therefore, the temperature sensor transfer function in Equation (12) is:

(12) $$\frac{T_{sen}\left(s\right)}{T(s)}=\frac{1}{6.218s+1}$$

**Figure 11:**
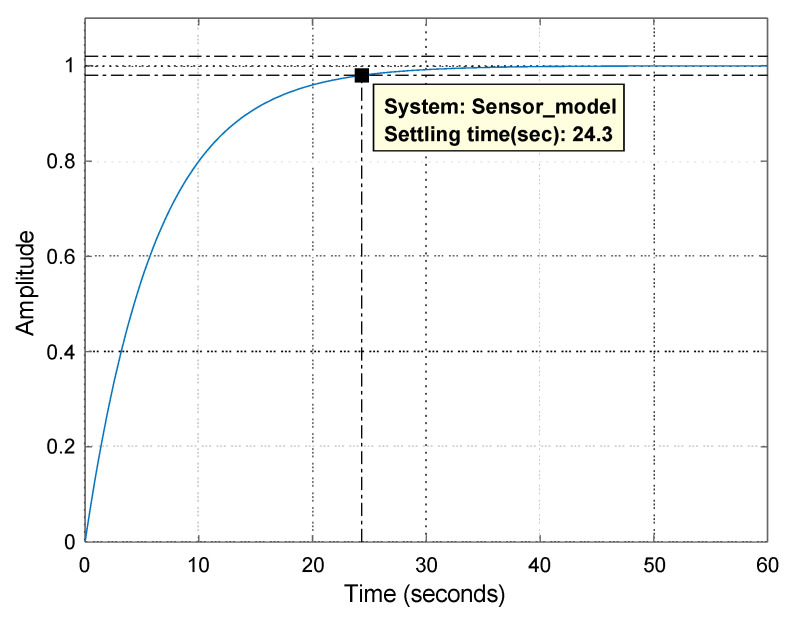
Step response of the temperature sensor transfer function.

The step response, Figure 11, of the sensor model shows that the response time is about 25 s and the steady-state gain is equal to one.

### 6.2. PID Controller

**Figure 12:**
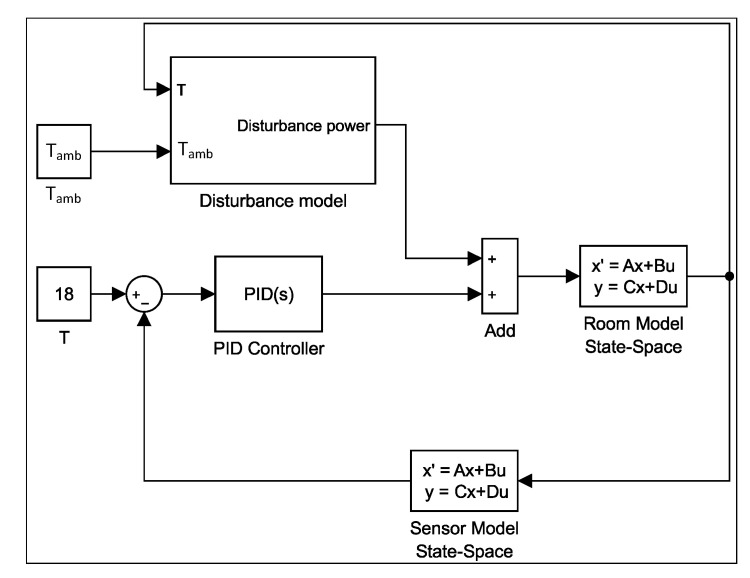
PID closed-loop control system in Simulink.

Now, the desired room temperature was set to 18 °C and the ambient temperature is 36 °C. The PID controller parameters, shown in Table 4, were chosen using the Ziglar–Nicholas tuning method to achieve a reasonable performance. The closed-loop system built in MATLAB Simulink is presented in Figure 12.

**Figure 13:**
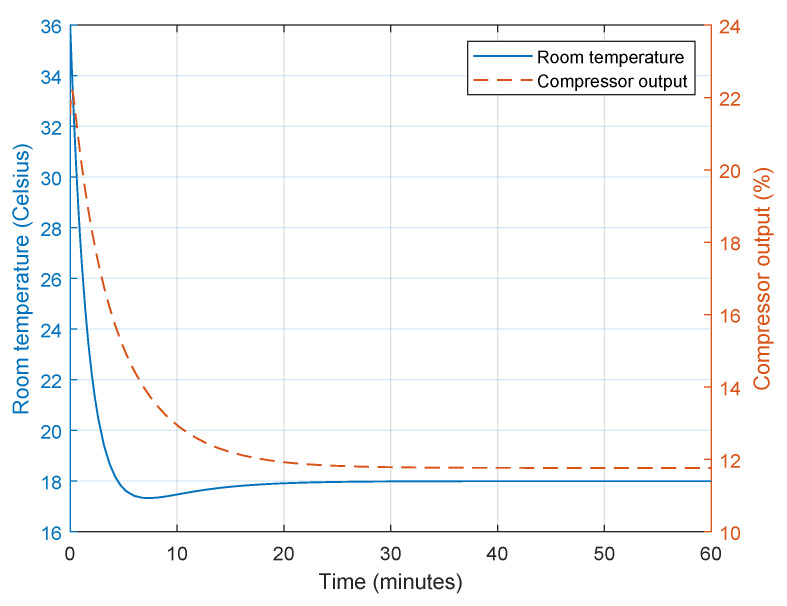
Room temperature response and compressor output.

As shown in Figure 13, the compressor initially operates at 22% of its maximum power when the difference between the room temperature and ambient temperature is greatest, and this ratio is reduced to about 12% when the room temperature reaches the desired value after 15 min. This is because the AC frequency converter adjusts the compressor output to the room temperature by controlling the inverter.

**Figure 14:**
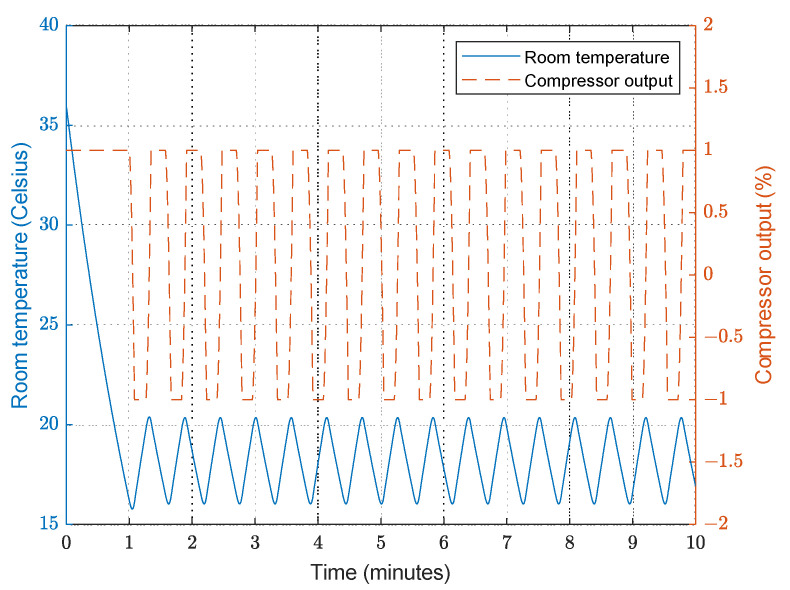
Room temperature response and compressor output with fixed-frequency AC.

When the fixed-frequency AC system is used, the compressor is controlled to be either On or Off, allowing the room temperature to rise quickly; however, this behaviour is not comfortable for the people in the room, as the compressor is either always On or Off, and the temperature is not maintained accurately, as shown in Figure 14.

This is not the real working scenario (the AC is only in cooling mode), but for the purposes of our study, we will accept the idea that the AC switches to heating mode when the temperature is lower than expected.

### 6.3. Smart FPN-Based Framework Implementation

To illustrate the efficiency of the proposed FPN-based framework, we conducted an experiment in a room of 16 m^2^ that can accommodate several users. The developed system is a simulation of a real environment coded in MATLAB. The system mimics an AC unit with a PID controller and sensors to collect the necessary data. Our main goal throughout the experiment is to reduce energy consumption. To achieve this, we aimed to reach the optimum response time as a performance indicator. We used a face detection algorithm to identify users in the room and adjust the temperature according to their preferences. The FPN is used to obtain user preferences and send this information to the PID controller to adjust the AC settings. Using the FPN model allowed us to verify the flow of data between all system elements and was useful in dealing with linguistic data such as temperature expressed as low, normal, and high. The maximum power of the compressor was set to 850 W. To prove the effectiveness of the FPN-based framework in terms of time, energy consumption and user comfort, we compared its performance to two other control methodologies:

- On/Off controller;
- The PN framework [11].

#### 6.3.1. User Identification

To simulate a realistic user identification experience, we utilized an image of the user who is expected to enter the room. A database was created containing pictures of users along with their corresponding temperature preferences. When a user enters the room, a face detection method is utilized to detect the faces in the image. The Viola–Jones algorithm was selected as the primary face detection technique. Subsequently, features were extracted from the images, and then compared to the database images. The user identification phase thus consists of three steps:

1. Face identification (Viola–Jones);
2. Feature extraction;
3. User recognition.

In this experiment, the system performance was evaluated with the participation of three users. Each user had a different preferred temperature, with

- User 1 preferring 20 °C;
- User 2 preferring 23 °C;
- User 3 preferring 26 °C.

The default room temperature was set to 30 °C, and the model was run for one hour. At the beginning of the trial, the system was turned Off and put into Standby mode, waiting for a user to enter the room. The initial room temperature was set to 36 °C, and gradually decreased as the system begins to regulate the temperature towards 30 °C.

#### 6.3.2. PID Controller

**Figure 15:**
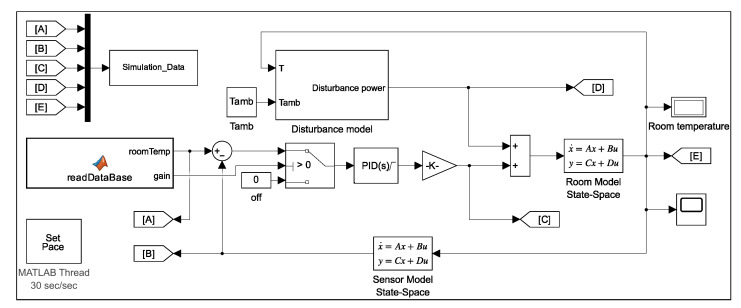
Simulink simulation of the temperature model.

The first part, when using the framework, is to read the user’s database and send control signals to the switch that turns the PID controller On and Off. In the actual experiment, the PID controller takes the desired temperature from the sensor and calculates the actuator output by summing the proportional, integral and derivative responses. The switch keeps the PID controller running when the system is operating or in a stable state. Figure 15 shows the Simulink diagram that displays the system components in the simulation experiment.

#### 6.3.3. Temperature Adjustment Using PN

**Figure 16:**
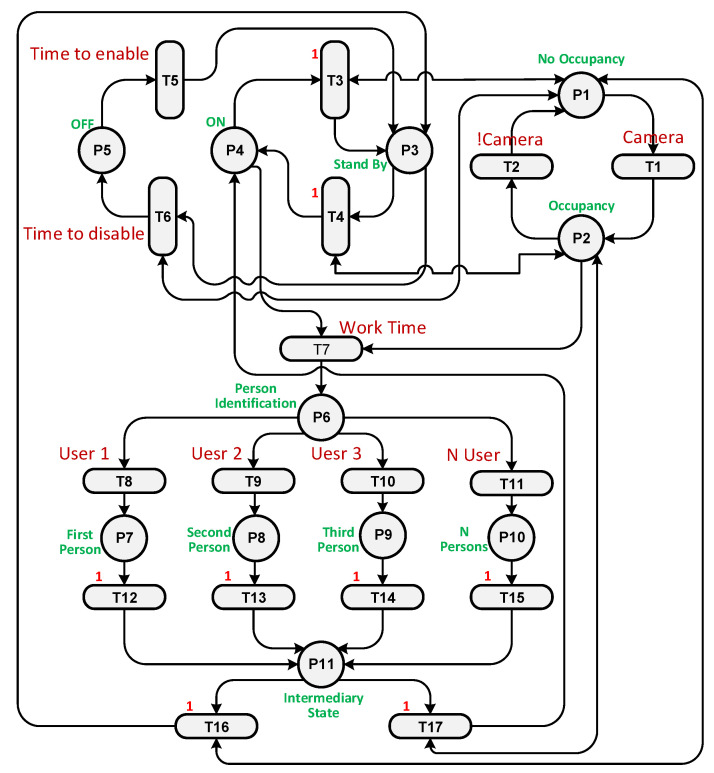
The PN structure.

We built a PN by setting transitions and places based on their functions and connecting them with arcs. Table 5 shows the places and transitions used in the PN in Figure 16. For example, places P1 and P5 represent the state when the system is Off. They have one token if the system is Off or at the beginning of the experiment. Tokens are moved from places and transitions through arcs during the completion of each cycle of the testing experiment. To facilitate monitoring, we added a graphical user interface (GUI) to display the token flow within the PN.

A loop was run until the end of the simulation time, and at each loop iteration, the transitions were checked to see which one was enabled and active. Transition T1 was enabled as P1 has a token, but it was inactivated as the sensor had not detected a user. T5 is responsible for enabling the system if the current time is later than the start time. T6 is fired when the Off time arrives. If the current time is greater than the start time and less than the Off time, it is the working time, so T7 must be activated and fired. Each place and transition has a specific function that works in the cycle.

**Figure 17:**
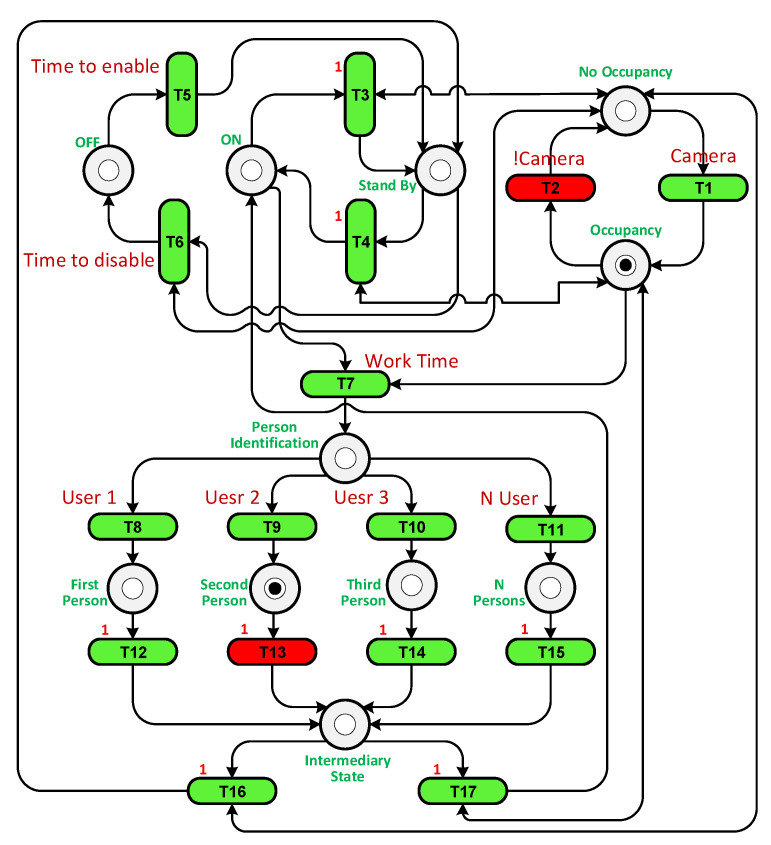
The PN structure with user 2 in the room.

**Figure 18:**
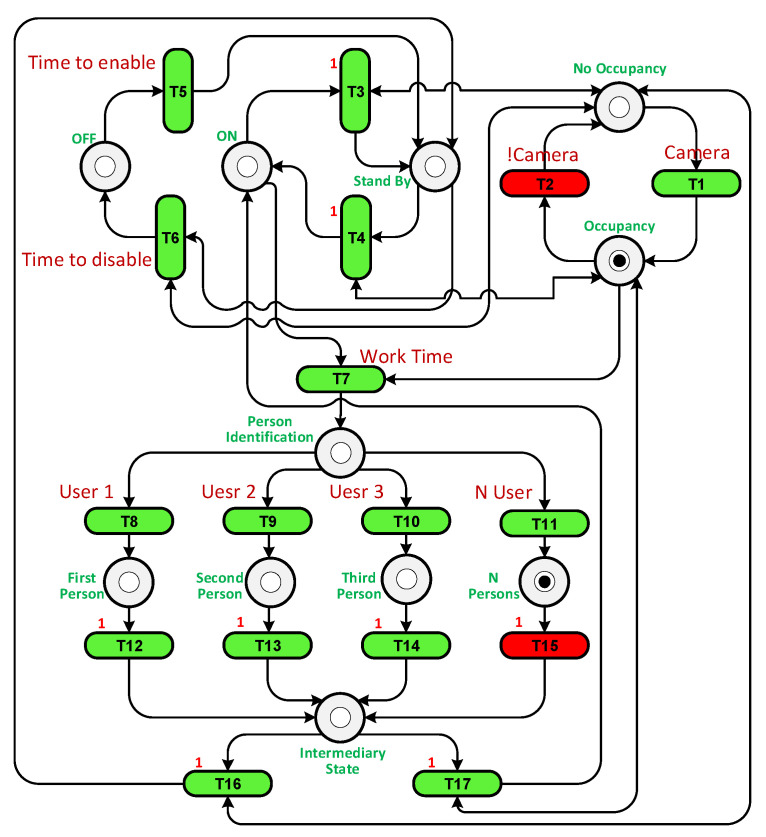
The PN structure with user 2 and 3 in the room.

To illustrate how the PN controller works, two scenarios were considered: when only user 2 is present in the room and when users 2 and 3 are present together. Unfortunately, in both cases using the On/Off controller, the system was unable to reach the precise temperature desired by the users, leading to discomfort. The purpose of the PN experiment was to increase temperature fluctuations in an attempt to achieve the exact temperature required. The PN structures for both scenarios can be observed in Figure 17 and Figure 18. In the first case, transition T13 is triggered when user 2 is detected in the room. In the second case, transition T15 is fired when there are multiple users present. Transition T2 is activated when the camera identifies the presence of a user in the room.

#### 6.3.4. Temperature Adjustment Using the FPN

Finally, we tested the application of the FPN in the same scenarios as above. FL used linguistic terms to express the output temperature and provide a percentage of membership in each temperature category (low, normal, or high). In a traditional PN, a token may or may not be present in a place, and transitions can only be fired if the required number of tokens are present in the input. In contrast, in an FPN, tokens can have different degrees of membership in a place, representing the degree of satisfaction of a particular condition. Transitions can then be fired according to the membership degree of the tokens, allowing more flexible modelling of the system behaviour.

A specialist’s experience is adopted in a controller by defining the best and most appropriate fuzzy rules and membership functions to handle the input and output signals. In this work, we merged two concepts: the PN model and the FL controller to build a high-level controller. This controller should maintain the room temperature as preferred by the person present in the room, and reduce energy consumption as much as possible.

**Figure 19:**
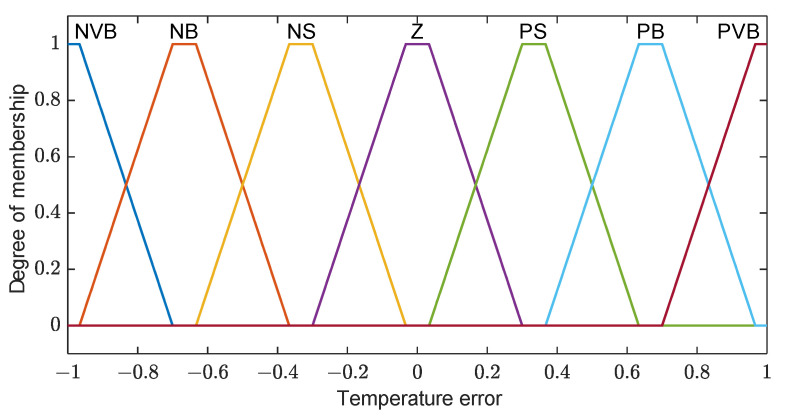
Temperature error membership functions.

Membership functions are set based on our knowledge to classify the temperature error in degrees Celsius as follows: Zero (Z); negative small (NS); negative big (NB); negative very big (NVB); positive small (PS); positive big (PB); positive very big (PVB), as shown in Figure 19.

**Figure 20:**
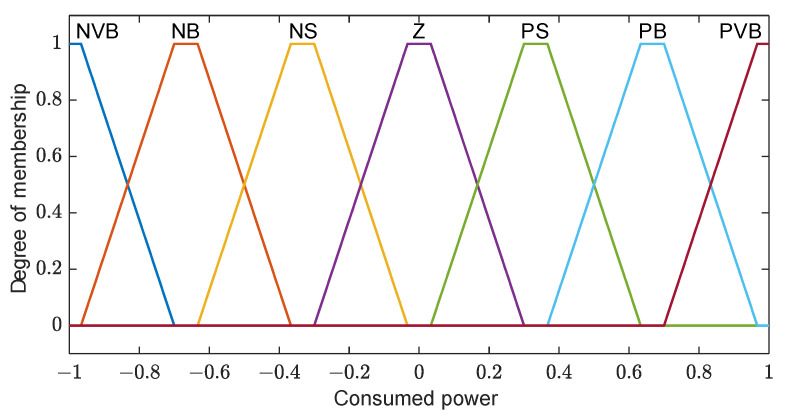
Consumed energy membership functions.

The primary objective of a controller is to ensure that the desired output is achieved in response to a given input. In the context of this work, the controller is responsible for adjusting the room temperature by providing appropriate commands to the AC compressor, which can either cool down or heat up the room. The energy consumption of the AC unit is represented by a membership function, as illustrated in Figure 20. The negative and positive segments represent the cooling and heating states, respectively.

**Figure 21:**
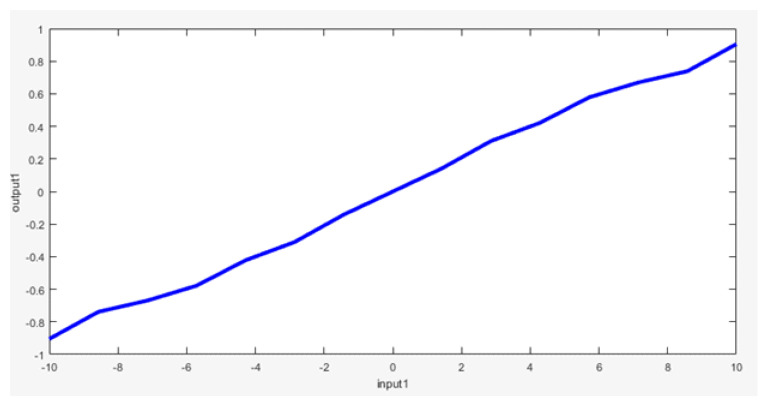
Relation between the input and output.

Fuzzy relations between the input and output are set to be as simple as possible, so it can be represented as line with a slope of 0.1, as shown in Figure 21.

The linear relationship between the temperature error and compressor output implies that a larger temperature error corresponds to a higher compressor power. For instance, when the temperature error is 6 °C, the compressor power is approximately 0.6% of its maximum capacity. To operate the AC unit at its maximum power, the temperature error needs to exceed 10 °C. The following rules are employed in this context:

1. If the temperature error is NVB, then the compressor output is NVB;
2. If the temperature error is NB, then the compressor output is NB;
3. If the temperature error is NS, then the compressor output is NS;
4. If the temperature error is Z, then the compressor output is Z;
5. If the temperature error is PS, then the compressor output is PS;
6. If the temperature error is PB, then the compressor output is PB;
7. If the temperature error is PVB, then the compressor output is PVB.

**Figure 22:**
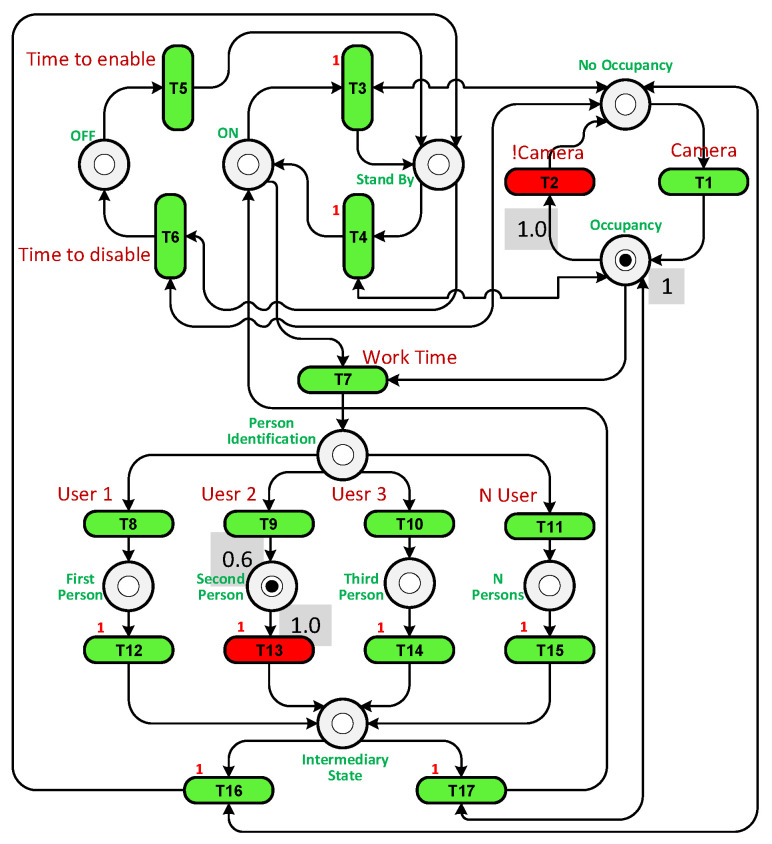
The FPN structure with user 2 in the room.

**Figure 23:**
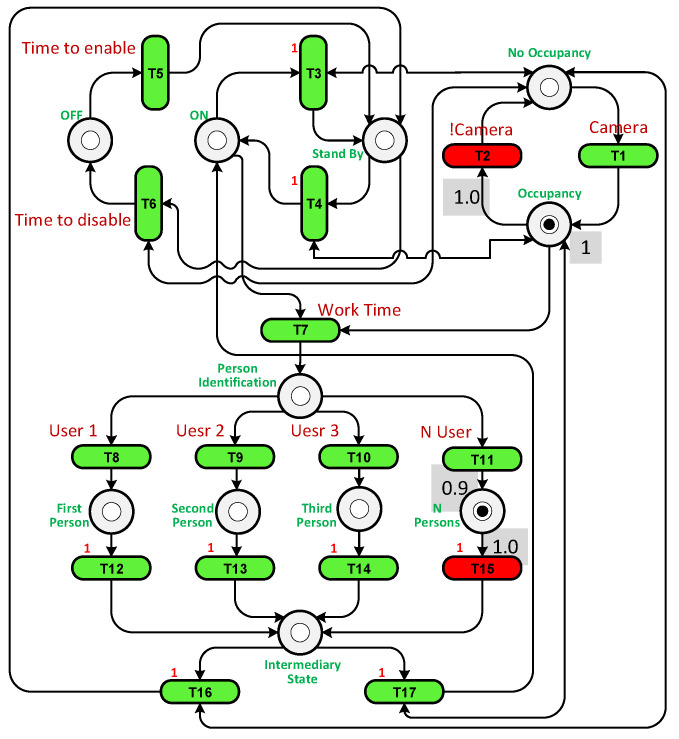
The FPN structure with user 2 and 3 in the room.

The FPN structures for both scenarios are depicted in Figure 22 and Figure 23. The utilization of FPNs leads to a reduction in energy consumption. By default, the temperature is set to its normal value. When user 2 enters the room, the fuzzy system adjusts the temperature to 60% (see Figure 22, T9) of the normal value, and when both user 2 and 3 enter the room, the temperature is set to 90% (see Figure 23, T11) of the normal value.

**Figure 24:**
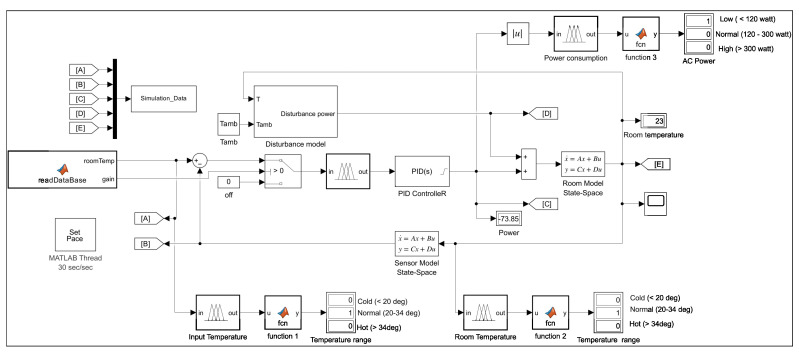
Simulink simulation of the FPN model when user 2 is in the room.

Figure 24 shows the Simulink diagram that displays the system components in the simulation experiment.

## 7. Energy Consumption Analysis

In this test, we sought to analyse the performance of the system over the course of a day. The simulation started at 12:00 and lasted 24 h to measure the energy consumption, temperature control, and compressor power. Three models were tested, namely, the PN model, the FPN model and the On/Off model. The room was occupied as shown in the Table 6.

**Figure 25:**
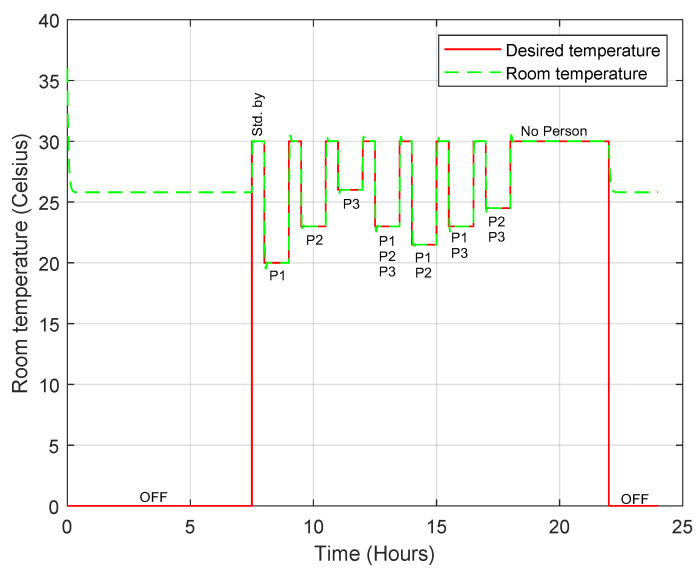
Room temperature profile when the convertible-frequency compressor is used in the case of the PN model.

**Figure 26:**
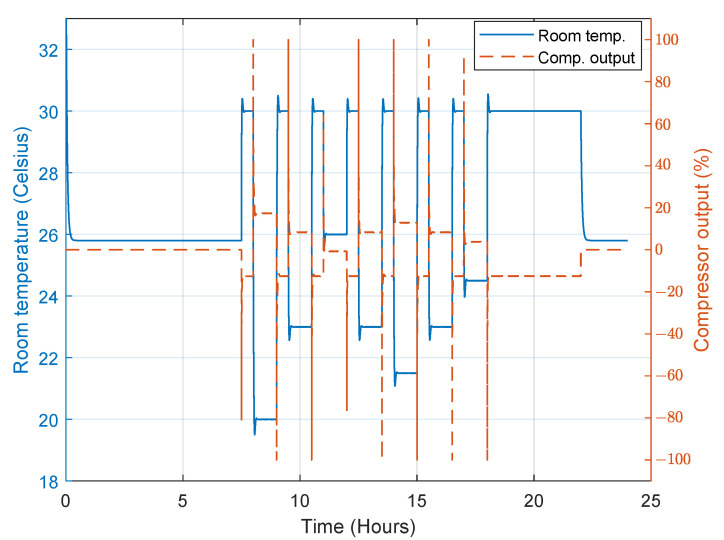
Compressor output when the convertible-frequency compressor is used in the case of the PN model.

The first person’s preferred temperature is 20 °C, the second person’s preferred temperature is 23 °C and the third person’s preferred temperature is 26 °C. In this simulation, we assume that the outdoor room temperature is 36 °C. When there is more than one person in the room, the desired temperature is set to the average of the preferred temperatures. When no one is inside the room, a standby temperature of 30 °C is set. Figure 25 shows the temperature profile during the simulation when the PN model is used, and it is clear that the controller (convertible-frequency mode) is able to maintain the desired temperature successfully and accurately. The compressor power varies continuously from 0 W to the maximum power depending on the required power dissipation. The total energy consumption in this mode is about 210 Wh, which is low compared to the 850 Wh of the AC, as shown in Figure 26.

**Figure 27:**
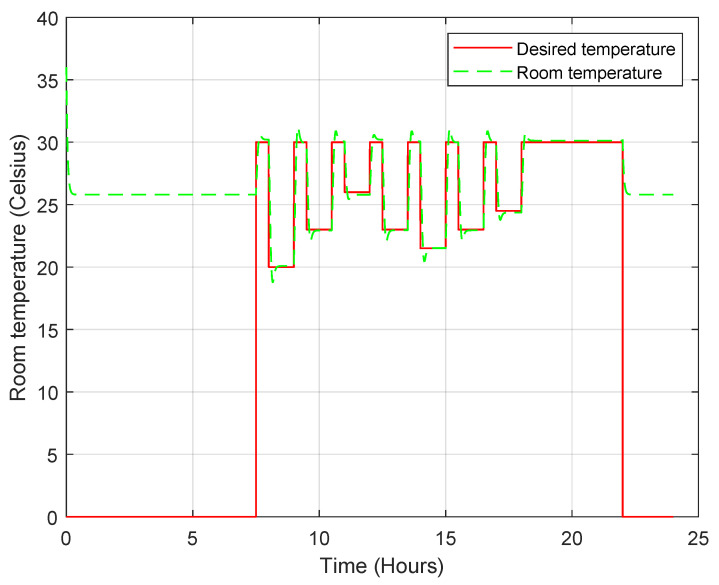
Room temperature profile when the convertible-frequency compressor is used in the case of the FPN model.

**Figure 28:**
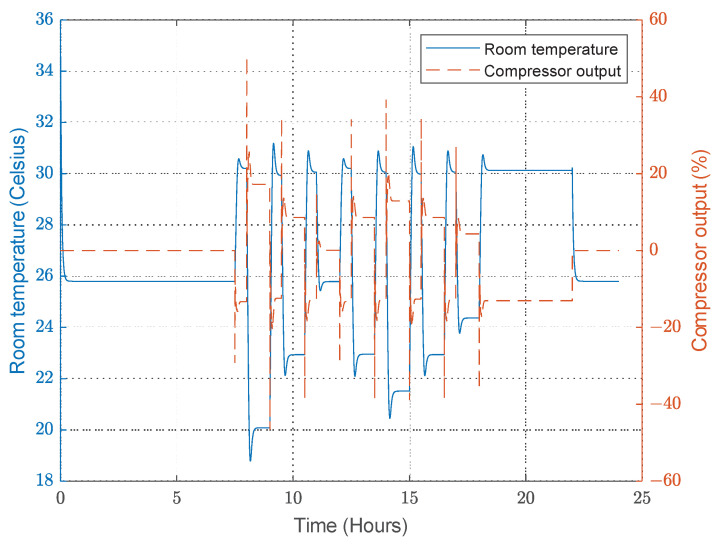
Compressor output when the convertible-frequency compressor is used in the case of the FPN model.

The FPN model behaves similarly to the PN model in that it uses the designer’s experience to decide what action to take based on the situation. Therefore, the controller follows the commands of the FPN model to maintain the desired temperature. Figure 27 shows that the temperature of the room is precisely controlled, similar to the PN model, but the main difference is the total energy consumption. The FPN model can perform the same test scenario using only 45 Wh.The steady-state temperature error is about 0.5 °C, larger than the error obtained in the PN model, as shown in Figure 28, because the fuzzy rules interfere in the control and affect the PID controller input.

**Figure 29:**
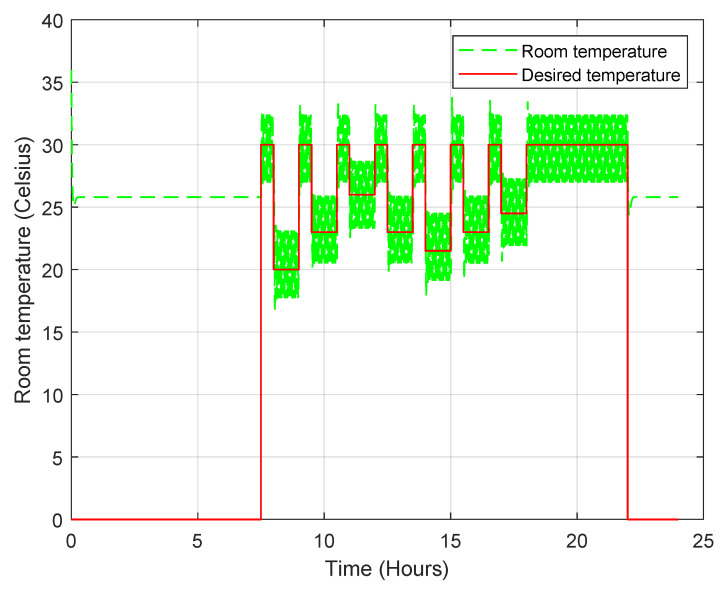
Room temperature profile when the fixed-frequency compressor is used in the case of the On/Off switching model.

**Figure 30:**
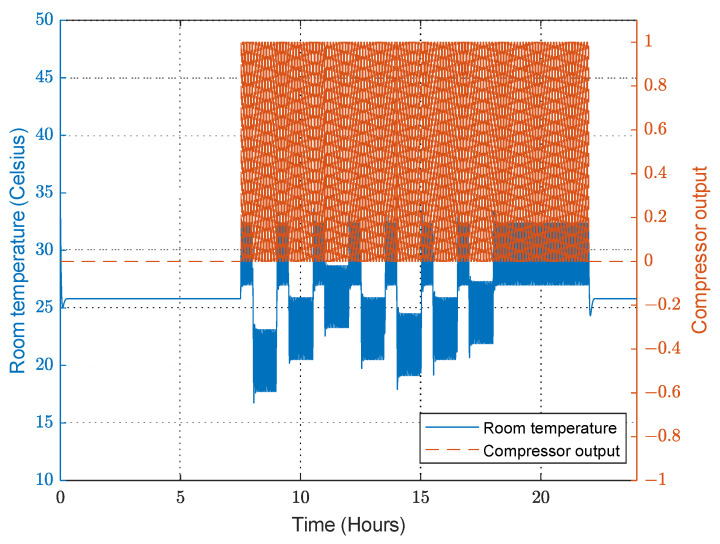
Compressor output when the fixed-frequency compressor is used in the case of the On/Off switching model.

A second similar simulation was performed with a fixed-frequency compressor; as shown in Figure 29, the room temperature is regulated approximately around the desired temperature because the compressor is either On (room temperature decreases) or Off (room temperature increases) and it is difficult to achieve zero error. The total energy consumption in this mode is around 345 Wh, corresponding to around 40% of the rated AC power. The AC power is always On and Off, which is inefficient and uncomfortable for the person in the room. The steady-state temperature error shown in Figure 30 is large compared with the FPN and PN models.

From the results presented above, we find that the error rate when using the PN is lower than the error rate with the FPN. However, it is important to consider the energy consumption. The difference in energy consumption between the PN and FPN is noticeable and large compared to the difference in the temperature error, with no effect on user comfort and flexibility.

The results presented in Table 7 compare the energy consumption and temperature error for the different compressor modes in the implemented models. The On/Off switching model, which utilizes a fixed-frequency compressor, shows a temperature error of approximately ±2.5 °C, with an energy consumption of 345 Wh. The PN model, incorporating a convertible-frequency compressor, demonstrates a significantly reduced temperature error of less than 0.1 °C, with a energy consumption of 210 Wh. The FPN model, also employing a convertible-frequency compressor, achieves further improvement with a temperature error of approximately ±0.5 °C, resulting in a remarkably reduced energy consumption of only 45 Wh. These findings indicate that both the PN and FPN models outperform the On/Off switching approach in terms of temperature control accuracy and energy efficiency. Furthermore, the FPN model demonstrates superior performance, effectively reducing energy consumption compared to the PN model. Overall, the integration of FL into the PN framework enhances the temperature control precision and energy efficiency, with the FPN model yielding the most favourable results.

## 8. Advantages of the Proposed Framework

The advantages of the proposed method can be summarized as follows:

- Enhanced user comfort: Our framework puts the user first, considering the individual’s preferences, perceptions and choices. By integrating fuzzy concepts such as “good temperature”, “warm temperature” or “low temperature”, the system accounts for subjective and variable preferences, thus providing a more personalized and adaptable solution. This integration makes it possible to model uncertainty and imprecision, improving user comfort and satisfaction.
- Energy efficiency: The proposed approach optimizes energy consumption by using degrees of cure within the FPN. This optimization, combined with the ability to communicate individual comfort requirements, leads to significant improvements over traditional fixed temperature control systems. By striking a balance between energy efficiency and occupant comfort, the proposed method offers a more efficient, user-centric temperature control system.
- Flexibility and adaptability: The integration of FPNs enables different user preferences to be flexibly modelled. The framework can adapt to changing user needs and preferences, resulting in a more adaptable and versatile temperature control system. Furthermore, the system is highly responsive to environmental changes. The framework’s flexibility also allows rapid adjustments to circumstances or variables, reinforcing its effectiveness in office temperature control. When several users are present, the framework accounts for the desired average temperatures, further enhancing its adaptability.

By considering fuzzy concepts and optimizing energy consumption within the FPN framework, our proposed method provides a more personalized, adaptable and energy-efficient temperature control solution. These advances are aimed at improving user comfort and satisfaction, as well as overall system efficiency.

## 9. Conclusions

The proposed framework utilizes FL to enhance the performance of the PN-based control system for regulating building temperature and optimizing energy usage. By implementing a facial recognition system to identify individuals and adjust the temperature according to their preferences, a comfortable indoor environment is achieved. A database is created to store user information, including names, ID numbers, temperature preferences, and facial features. The system is controlled using an FPN controller, effectively managing different operational modes based on user presence.

Initially, the system remains in an Off state until the designated start time, entering Standby mode with an ambient temperature set to 30 °C to conserve energy. Once a registered user is recognized, the temperature is adjusted to their preferred level. In cases where multiple users are present, the system calculates the average of their preferred temperatures. The FPN controller ensures that the AC unit is turned Off when a predefined time limit is reached.

Simulations conducted using two types of compressors, a convertible-frequency AC compressor and a fixed-frequency compressor, demonstrate the effectiveness of the proposed system. Evaluation of the system reveals significant energy savings, with the FPN model and the convertible-frequency compressor reducing energy consumption by 94% (45 Wh). When only using the PN model a 25% reduction in energy consumption (210 Wh) is achieved, while employing a fixed-frequency compressor leads to a 40% increase in energy usage (345 Wh). The incorporation of FL into the PN model proves highly advantageous, effectively reducing energy consumption by half.

In conclusion, this research presents a framework that effectively utilizes FL to improve the performance of a PN-based control system for building temperature regulation and energy optimization. The implementation of a facial recognition system and user preferences further enhances user comfort. Future research should focus on exploring the use of alternative control algorithms within the proposed framework, or studying the impact of external factors on energy efficiency. By addressing these areas, new advances can be made in the development of energy-efficient building control systems.

## Figures and Tables

**Table 1:** System parameters.

Symbol	Refer To
*T*	Indoor temperature
$T_{amb}$	Outdoor temperature
$(dQ_{abs})/dt$	Removed heart overflow
*C*	Total specific heat capacity
*M*	Thermal mass
*A*	Indoor surface area
*X*	Outdoor wall thickness
*k*	Thermal conductive coefficient

**Table 2:** Room model parameters.

Parameter	Value	Unit
Ambient Temperature	$T_{amb}$ = 36	°C
Desired Room Temperature	$T_{des}$ = 20	°C
Number of Persons Indoor	n=1	#
Room Volume	V=4∗4∗3	m^3^
Air Density	ρ=1.184	kg/m^3^
Thermal Mass	M=n∗85+Rou∗V	Kg
Total Specific Heat Capacity	C=20	J/Kg/C
Exchanging Air Flow	$m^{\prime }$ = 1/30	m^3^/s
Heat Capacity at Constant Pressure	$C_{p}$ = 1	J/Kg/K
Thermal Conductivity Coefficient	k=0.2	W/m/K
Indoor Surface Area	A=4∗4	m^2^
Outdoor wall thickness	X=0.25	m

**Table 3:** Sensor parameters and characteristics.

Parameter	Value	Units
Sensor Mass	$M_{sen}$ = 0.05	Kg
Sensor Heat Capacity	$C_{sen}$ = 0.385	KJ/kg/K
Sensor convection coefficient	$h_{sen}$ = 13.14	W/m^2^/k
Sensor area	$A_{sen}=3.36*10^{-4}$	m^2^
Maximum Compressor Power	850	W

**Table 4:** PID parameters.

KP	KI	KD
10	$M_{sen}$ = 0.1	0

**Table 5:** Place list in the PN.

Place Name	Transition Name	Antecedent/Consequent
P1	T1	No occupancy in the room
P2	T2	An occupant in the room
P3	T3	Air conditioner on Standby
P4	T4	Air conditioner is On
P5	T5	Air conditioner is Off
P6	T6	Person identification
P7	T7	First person
P8	T8	Second person
P9	T9	Third person
P10	T10	More than one person
P11	T11	Intermediary state

**Table 6:** Time schedule for the working scenario.

From	To	Action
12:00 AM	7:30 AM	System is off
7:30 AM	8:00 AM	System is in Standby mode
8:00 AM	9:00 AM	First person is in the room
9:00 AM	9:30 AM	No people are in the room
9:30 AM	10:30 AM	Second person is in the room
10:30 AM	11:00 AM	No people are in the room
11:00 PM	12:00 PM	Third person is in the room
12:00 PM	12:30 PM	No people are in the room
12:30 PM	1:30 PM	All three people are in the room
1:30 PM	2:00 PM	No people are in the room
2:00 PM	3:00 PM	First and second person are in the room
3:00 PM	3:30 PM	No people are in the room
3:30 PM	4:30 PM	First and third person are in the room
4:30 PM	5:00 PM	No people are in the room
5:00 PM	6:00 PM	Second and third person are in the room
6:00 PM	10:00 PM	No people are in the room
10:00 PM	12:00 AM	System is off

**Table 7:** Energy consumption and temperature error results for the three compressor modes.

Model	Temperature Error	Energy Consumption	Compressor Mode
On/Off switching	±2.5	345 Wh	Fixed-Frequency
PN	<0.1	210 Wh	Convertible-Frequency
FPN	±0.5	45 Wh	Convertible-Frequency

## References

[1] Mezghani I., Haddad H.B. (2017). Energy consumption and economic growth: An empirical study of the electricity consumption in Saudi Arabia. Renew. Sustain. Energy Rev..

[2] Agboola M.O., Bekun F.V., Joshua U. (2021). Pathway to environmental sustainability: Nexus between economic growth, energy consumption, CO2 emission, oil rent and total natural resources rent in Saudi Arabia. Resour. Policy.

[3] IEA KSA Electricity Consumption IEA Report, 2022. https://www.iea.org/countries/saudi-arabia.

[4] Petri C. (1962). Kommunikation mit Automaten.

[5] Giua A., Silva M. (2018). Petri nets and automatic control: A historical perspective. Annu. Rev. Control.

[6] Murata T. (1989). Petri nets: Properties, analysis and applications. Proc. IEEE.

[7] Jensen K., Kristensen L.M. (2015). Colored Petri nets: A graphical language for formal modeling and validation of concurrent systems. Commun. ACM.

[8] Jensen K., Kristensen L.M. (2009). Coloured Petri Nets: Modelling and Validation of Concurrent Systems.

[9] Miyagi P.E., Villani E., Gustin G., Maruyama N., Santos Filho D.J.d. (2002). Petri net approach for modelling system integration in intelligent buildings. J. Braz. Soc. Mech. Sci..

[10] Ozkan H.A. Petri net modelling of smart home appliances. Proceedings of the 2017 International Conference on Smart Systems and Technologies (SST).

[11] Bouazza K.E., Deabes W. (2019). Smart Petri nets temperature control framework for reducing building energy consumption. Sensors.

[12] Subahi A.F., Bouazza K.E. (2020). An intelligent IoT-based system design for controlling and monitoring greenhouse temperature. IEEE Access.

[13] Ajao L.A., Agajo J., Umar B.U., Agboade T.T., Adegboye M.A. Modeling and Implementation of Smart Home and Self-control Window using FPGA and Petri Net. Proceedings of the 2020 IEEE PES/IAS PowerAfrica.

[14] Yang C.Y., Lin Y.N., Shen V.R., Shen F.H., Jheng W.S. (2023). A Novel IoT-Enabled System for Real-Time Monitoring Home Appliances Using Petri Nets. TechRxiv.

[15] Zadeh L.A. (1965). Fuzzy sets. Inf. Control.

[16] Dubois D., Esteva F., Godo L., Prade H., Gabbay D.M., Woods J. (2007). Fuzzy-Set based logics—A history-oriented presentation of their main developments. Handbook of the History of Logic.

[17] Lei T., Wang Y., Jin X., Min Z., Zhang X., Zhang X. (2022). An optimal fuzzy logic-based energy management strategy for a fuel cell/battery hybrid power unmanned aerial vehicle. Aerospace.

[18] Sarojini R.K., Palanisamy K., De Tuglie E. (2022). A fuzzy logic-based emulated inertia control to a supercapacitor system to improve inertia in a low inertia grid with renewables. Energies.

[19] Zimmermann H.J. (1978). Fuzzy programming and linear programming with several objective functions. Fuzzy Sets Syst..

[20] Zhou K.Q., Zain A.M. (2016). Fuzzy Petri nets and industrial applications: A review. Artif. Intell. Rev..

[21] Lipp H.P. (1983). The Application of a Fuzzy Petri Net for Controlling Complex Industrial Processes. IFAC Proc. Vol..

[22] Cardoso J., Valette R., Dubois D. (1996). Fuzzy Petri nets: An overview. IFAC Proc. Vol..

[23] Liu H.C., You J.X., Li Z., Tian G. (2017). Fuzzy Petri nets for knowledge representation and reasoning: A literature review. Eng. Appl. Artif. Intell..

[24] Yakrangi O., Saltarén Pazmiño R.J., Cely J.S., Rodríguez A., García Cena C.E., San Segundo Carrillo P., De La Cueva J., Shapiro A. (2021). An intelligent algorithm for decision making system and control of the GEMMA guide paradigm using the fuzzy petri nets approach. Electronics.

[25] Jiang W., Zhou K.Q., Sarkheyli-Hägele A., Zain A.M. (2022). Modeling, reasoning, and application of fuzzy Petri net model: A survey. Artif. Intell. Rev..

[26] Bause F., Kritzinger P.S. (1998). Stochastic Petri Nets: An Introduction to the Theory. Sigmetrics Perform. Eval. Rev..

[27] Zadeh L.A. (2015). Fuzzy logic—A personal perspective. Fuzzy Sets Syst..

[28] Precup R.E., Preitl S., Petriu E., Bojan-Dragos C.A., Szedlak-Stinean A.I., Roman R.C., Hedrea E.L. (2020). Model-based fuzzy control results for networked control systems. Rep. Mech. Eng..

[29] Belman-Flores J.M., Rodríguez-Valderrama D.A., Ledesma S., García-Pabón J.J., Hernández D., Pardo-Cely D.M. (2022). A Review on Applications of Fuzzy Logic Control for Refrigeration Systems. Appl. Sci..

[30] Omarov B., Anarbayev A., Turyskulov U., Orazbayev E., Erdenov M., Ibrayev A., Kendzhaeva B. (2020). Fuzzy-PID based self-adjusted indoor temperature control for ensuring thermal comfort in sport complexes. J. Theor. Appl. Inf. Technol..

[31] Liu F., Heiner M., Gilbert D. (2020). Fuzzy Petri nets for modelling of uncertain biological systems. Brief. Bioinform..

[32] Bobryakov A., Prokopenko S., Misnik A., Krutalevich S. (2021). Modeling of industrial and technological processes in complex systems based on neuro-fuzzy Petri nets. J. Phys. Conf. Ser..

[33] Taj S.M., Kumaravel A. (2015). Survey on fuzzy Petri nets for classification. Indian J. Sci. Technol..

[34] Fay A. (2000). A fuzzy knowledge-based system for railway traffic control. Eng. Appl. Artif. Intell..

[35] Yang H.T., Huang C.M. (2002). Distribution system service restoration using fuzzy Petri net models. Int. J. Electr. Power Energy Syst..

[36] Sharma R.K., Kumar D., Kumar P. (2008). Predicting uncertain behavior of industrial system using FM—A practical case. Appl. Soft Comput..

[37] Ting Y., Lu W.B., Chen C.H., Wang G.K. (2008). A fuzzy reasoning design for fault detection and diagnosis of a computer-controlled system. Eng. Appl. Artif. Intell..

[38] Borase R.P., Maghade D., Sondkar S., Pawar S. (2021). A review of PID control, tuning methods and applications. Int. J. Dyn. Control.

[39] Díaz-Rodríguez I.D., Han S., Bhattacharyya S.P. (2019). Analytical Design of PID Controllers.

[40] Al-Moslmi T., Ocaña M.G., Opdahl A.L., Veres C. (2020). Named entity extraction for knowledge graphs: A literature overview. IEEE Access.

[41] Abdel-Hakim A.E., El-Saban M. Face authentication using graph-based low-rank representation of facial local structures for mobile vision applications. Proceedings of the IEEE International Conference on Computer Vision Workshops (ICCV Workshops).

[42] Cheng C.C., Lee D. (2014). Smart Sensors Enable Smart Air Conditioning Control. Sensors.

